# From beach to bedside: lessons from the Australian treatment system for dealing with the challenges faced by the UK’s addiction medical workforce crisis

**DOI:** 10.1192/bji.2025.10091

**Published:** 2026-11

**Authors:** Emmert Roberts, Michael Farrell

**Affiliations:** 1 Dr, Senior Clinical Lecturer, National Addiction Centre, Institute of Psychiatry, Psychology and Neuroscience, King’s College London, London, UK; 2 Professor, Director, National Drug and Alcohol Research Centre, University of New South Wales, Sydney, Australia

**Keywords:** Addictions, workforce, addiction medicine, United Kingdom, Australia

## Abstract

Over the last decade in the UK the number of medically trained addiction specialists has halved, to record low numbers. Despite acknowledgment of this crisis by the UK Government in its recently published *10-Year Strategic Plan for the Addiction and Recovery Workforce*, psychiatric training remains the only available route to gain specialist medical accreditation in the treatment of addiction disorders. This article asks if it is time for the UK to embrace the creation of a new physician-led addiction medicine specialty akin to models developed in Australia and other peer countries.

In 2021, the landmark UK Independent Review of Drugs laid bare the stark picture of an addiction treatment system in a parlous state following sustained funding cuts, and crises in the training, recruitment and retention of the addiction workforce.^1^ Particular concerns highlighted declining numbers of medically trained specialists, the 2023 census reporting fewer than 0.5% (*n* = 56) of addiction staff as consultant-grade doctors, the number of full-time equivalent posts having halved over the previous decade.^2^ These trends are mirrored in the number of resident doctors gaining specialist accreditation each year, with fewer than a fifth of services able to provide a specialist training environment.^3^ Record numbers of drug and alcohol-related deaths are evidence of the negative consequences for the whole community.

This prompted the UK Department of Health and Social Care (DHSC) and NHS England to publish their *10-Year Strategic Plan for the Addiction Workforce*.^4^ While the report states that ‘building sustainable pipelines of regulated professionals into the sector is crucial’, it also acknowledges that it is ‘unlikely to meet targets’ related to increasing the number of medical specialists. The strategy’s conclusions declare that ‘It is important to note that there is no quick fix for the lack of medical workforce presence in the drug and alcohol treatment and recovery sector.’ Although there may be no instant solution, creative international responses have been developed, which do not appear to have warranted consideration in the 10-year strategic plan.

## Addiction psychiatry versus addiction medicine

Within the UK the only route to specialist medical accreditation in addiction treatment is through psychiatric training. Several other countries, most notably Australia, have developed dedicated programmes in addiction medicine to train consultant-grade physicians.^5^ These programmes have proved popular among resident doctors, led to expansions in clinical research and, crucially, provided an expanded pool of potential clinical leaders within multidisciplinary teams.^6^ Indeed, Australia’s equivalent workforce census in 2020 showed that 2% of addiction staff are specialist medical practitioners and the majority of workers (70%) report overall job satisfaction.^7^

The location of the medical addiction specialism solely within psychiatry in the UK has led to multiple challenges. Some residents with an interest in addiction are disinclined to pursue wider psychiatric training in order to latterly specialise in addiction,^8^ and changes to addiction service commissioning in 2010, devolving statutory responsibility to local authorities and introducing the possibility of competitive tendering, have resulted in fewer than a third of services being provided by National Health Service (NHS) mental health trusts, severely limiting the availability of training posts for psychiatric residents.^9^ The 10-year strategic plan does note these issues and empowers the NHS to secure additional training posts and explore opportunities to gain specialist accreditation through other routes, but these recommendations remain focused on psychiatry as the only path to specialist accreditation.^4^ The commissioning landscape in the UK is now such that addiction service contracts are frequently re-tendered every three to five years, leading to specialists having to upskill in commissioning and reporting practices and cultivate relationships with local authority commissioning staff to maintain contracts. This has led in turn to reductions in time devoted to direct clinical care, with the siloed nature of addiction commissioning limiting the development of innovative holistic healthcare services able to provide concurrent primary care, mental health and addiction support. This also limits the visibility of, and access to, medical addiction specialists within wider healthcare settings, a function which addiction medicine physicians routinely fulfil throughout Australia.^10^ Given the most common modifiable risk factors contributing to adult morbidity include alcohol use, tobacco use and hypertension, increasing the number of physicians with specialist addiction knowledge would appear pragmatic and serve to reduce both health inequities and the burden on acute healthcare services.^11^

## Is it time to act?

The Independent Review of Drugs made an additional recommendation to establish a ‘Centre of Addiction’, an institution that could bring the workforce together and enable development and delivery of accredited training, continuing professional development opportunities and a national leadership platform.^1,4^ The necessarily multidisciplinary focus of such a centre would appear in alignment with potential expansion of the specialist workforce to include addiction medicine physicians. While efforts were made in the early 2000s to establish such a specialty in the UK, they were met with scepticism, bureaucratic challenges and a reticence to cede addiction from psychiatric structures (M. Farrell. personal email communication, 2025). The extent of the workforce crisis, alongside the now well-developed and evidenced model in Australia, is such that problem, political and policy streams may currently be aligned to empower decision makers to enable the creation of a new specialty.

Patients with addiction disorders will always need doctors, and to let medical addiction expertise dwindle to potential non-existence given its current trajectory should not be an option. We need creative solutions and should therefore embrace and say a hearty g’day to the advent of addiction medicine in the UK.

## Data Availability

Data availability is not applicable to this article as no new data were created or analysed in this study.
